# Placenta Accreta Spectrum Disorders: Current Recommendations from the Perspective of Antenatal Imaging

**DOI:** 10.1055/s-0043-1770917

**Published:** 2023-07-21

**Authors:** Conrado Milani Coutinho, Alexia Viegas Georg, Ligia Conceição Assef Marçal, Albaro José Nieto-Calvache, Theophilus Adu-Bredu, Francesco D'Antonio, José Miguel Palacios-Jaraquemada

**Affiliations:** 1 Departament of Ginecology and Obstetrics, Hospital das Clínicas, Faculdade de Medicina de Ribeirão Preto, Universidade de São Paulo, Ribeirão Preto, SP, Brazil; 2 Clínica de Espectro de Acretismo Placentario, Hospital Universitario Fundación Valle del Lili, Cali, Colombia; 3 Universidad ICESI, Cali, Colombia; 4 Obstetrics and Gynecology Directorate, Komfo Anokye Teaching Hospital, Kumasi, Ghana; 5 Center for Fetal Care and High Risk Pregnancy, Department of Obstetrics and Gynecology, University of Chieti, Chieti, Italy; 6 CEMIC University Hospital and School of Medicine, Universidad de Buenos Aires, Argentina

## The Burden of a Previous Uterine Scar

Cesarean section (CS) is the most commonly performed surgical procedure in the United States (more than a million surgeries per year) and one of the most frequently performed procedures worldwide. 1 Although CS is a potentially life-saving procedure when correctly indicated, its worldwide use has steadily increased over the last decades (currently 21.1% globally, ranging from 5% in sub-Saharan Africa to 42.8% in Latin America and the Caribbean). Moreover, it will continue increasing worldwide (2030 projection: 28.5% globally, ranging from 7.1% in sub-Saharan Africa to 63.4% in Eastern Asia). 2 Dominican Republic, Brazil, Cyprus, Egypt and Turkey are the worldwide leaders, with CS rates ranging from 58.1% to 50.8%, respectively, which points to a worrying trend towards overmedicalization of childbirth and overuse of CS. 2 Other surgical procedures such as dilation, curettage, myomectomy, and surgical hysteroscopy are less frequent than CS. Still, due to the trend towards more advanced maternal age, the number of pregnant women previously submitted to these procedures also tends to increase. These data point to a growing number of pregnancies in surgically manipulated uteruses.

**Fig. 1:**
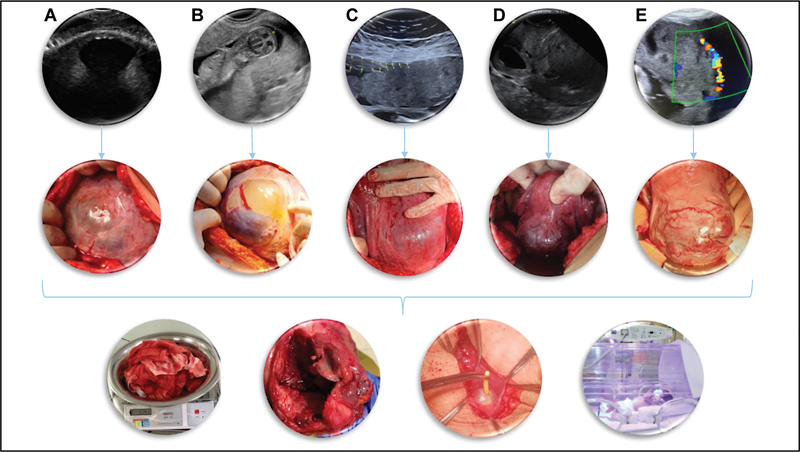
The broader spectrum of potential complications in pregnancies with prior uterine scars. On first and second lines, the respective ultrasound and surgical appearances of the following potential pregnancy abnormalities: **A** - myometrial dehiscence without overlying placenta; **B** - myometrial dehiscence with partial placental intrusion; **C** - myometrial dehiscence with complete placental intrusion; **D** - cesarean scar pregnancy; **E** - placenta accreta spectrum. Apart from case A, placenta previa is present on all other cases. On the lower line, potential perinatal adverse outcomes: major uterine bleeding, uterine rupture, unintentional bladder lesion, and neonatal complications of prematurity.

Pregnant women with previous uterine scars are at risk for increased morbimortality. Complications such as placenta previa, spontaneous uterine rupture, uterine dehiscence (with or without placental intrusion), cesarean scar pregnancy (CSP) and placenta accreta spectrum disorders (PAS) are associated with potentially life-threatening uterine bleeding, extra-uterine lesions and preterm delivery ( Figure 1 ). 3

A previous CS increases up to 60% the risk for placenta previa at delivery (approximate incidence: 0.3-2%), with a dose-response pattern based on the number of previous surgeries. 4 The incidence of uterine rupture was estimated as being 5.1 per 10,000 in scarred and 0.8 per 10,000 in unscarred uteruses, with 72% occurring during spontaneous labor. 5 A retrospective cohort of 169,356 pregnancies in a high-risk tertiary hospital reported 0.1% cases of uterine disruption - 83% dehiscence and 17% complete uterine ruptures - the latter significantly more associated with adverse perinatal outcomes. All these pregnancies had previous CS, mainly by low transverse incisions (60%). 6 CSP was estimated to range from 1:1,800 to 1:2,216 pregnancies, 52% in women with only one previous CS. 7 A systematic review and meta-analysis reported that the median prevalence of placenta previa with PAS was 0.07%, with an incidence of PAS in women with placenta previa of 11.1%. More than 90% of PAS cases occurred in women with a previous CS and low-lying/placenta previa. 8 Based on its mounting incidence and potential impact on maternal-fetal mortality, current strategies for mitigating the risks of CSP/PAS must be discussed.

## Updates on CSP/PAS Pathophysiology

Although the pathophysiological reasons for some women having abnormal uterine healing after uterine surgical procedures are still not fully understood, this might be mainly related to individual factors leading to remodeling of a previously healthy myometrium and its substitution for uterine niches with defective decidua basalis and low residual myometrial thickness. When implantation occurs over or into these defective scars, the closer proximity of the placenta with larger superficial arteries, increased fibrinoid deposition between placental villi and the myometrial layer, and adhesions from previous surgical procedures are directly associate with the occurrence of adverse outcomes. 9 10

Then, it is essential to emphasize contemporary pathophysiological concepts with important practical applicability regarding pregnancies in a scarred uterus. Current evidence does not support the role of placental “cancer-like invasion” on the pathophysiology of CSP/PAS 9 ; Instead, the shared histopathology between CSP and PAS has been already demonstrated, 11 and recent prospective studies 12 13 support the primary role of an abnormal implantation into a previously scarred myometrium, its progressive dehiscence and uterine remodeling as the proxy for the continuum between CSP and PAS. Therefore, the existence of extrauterine placental “invasion” (invasive percreta 14 or the International Federation of Gynecology and Obstetrics - FIGO 3b 15 ) is being currently challenged. In reality, the challenging surgical dissection of dense adhesive disease seems to produce the lesions disrupting the serosa/scar shell observed on gross anatomy, which can lead the pathologist to an incorrect diagnosis of placental “invasion”. 12 13 Also, the absence of placental “invasion” does not lessen the severity of PAS, as the surgical difficulty generated by the hypervascularization and anatomical distortion from dense adhesions that involves adjacent organs are associated with potential life-threatening complications. More importantly, increase production of vascular growth factor induces the formation of a rich anastomotic pattern between vaginal, uterine, and vesical arteries, which represents a surgical challenge and constitutes the basis for the massive hemorrhagic risk of women affected by PAS. Whether the placenta reaches the deeper layers of the uterine wall is less important than the size and topography of the lesion in the uterus, which are related to the severity and the type of treatment required. 16 17 18

## The Importance of Antenatal Screening/Diagnosis for PAS and Surgical Planning

Antenatal recognition of CSP/PAS is crucial, because timely referral of suspected cases to specialized referral centers with multidisciplinary teams that manage these complex cases continuously is the key to reducing perinatal morbimortality. 19 A retrospective Latin American study reported that among 52 maternal deaths related to PAS, 40% did not have antenatal diagnosis and almost 46% were not evaluated in a PAS referral hospital before delivery. According to the authors, all maternal deaths were potentially preventable, 77% by low- to moderate-complexity interventions. 20 Therefore, regional PAS care pathways should be set up to ensure every pregnant women has access to qualified primary care ultrasound and, in case of a positive screening for CSP/PAS, prompt referral for a specialized diagnostic center should be made. Fetal medicine providers specialized in PAS will be responsible for defining whether the patient should be managed in a specially funded PAS referral center or a low-complexity hospital. 21 Regional PAS referral centers should be carefully identified by stakeholders based on quality markers in care, such as application of comprehensive care models, human and technological resources, surgical expertise, self-assessment and research output. 22 23 24

For screening purposes, all sonographers responsible for obstetrical ultrasounds should always ask themselves two questions: (1) is the placenta low-lying? (2) did the patient have a previous uterine surgery? If the answer is positive to both questions, the patient should be considered at risk for CSP/PAS at any gestational age and referred to a PAS specialized diagnostic center. 25 Screening for CSP should ideally be performed for all patients with previous CS between 6-9 gestational weeks, when the gestational sac is more related to the uterine scar niche than to the uterine cavity, resulting in better accuracy. 26 The reason for the higher accuracy of ultrasound in detecting CSP in the early compared to the late first trimester relies on the fact that with advancing gestation, the upper pole of the gestational sac grows towards the uterine fundus, thus making assessment of the relationship between the sac and the area of the prior CS scar more difficult to assess. If the patient is first seen for the 11-14 week scan, positive answers to both questions should trigger a referral to the specialized center. 27 CSP/PAS detection at the time of 11-14 weeks scan has been also reported in several large studies with good sensitivity and specificity. 28 Despite that, the role of first trimester ultrasound in the detection of CSP/PAS in terms of clinical and economic effects is far from established. For locations with more restricted access to ultrasound, a contingent screening strategy for placenta previa on the 18-24 week scan, with a reassessment of persistent low-lying/placenta previa between 32-34 weeks and referral of patients to referral centers with positive answers for both questions at this moment seems to be more cost-effective and very accurate. 25 29 The disadvantage of this last strategy is the loss of opportunity for counselling and early treatment of CSP cases, which are associated with fewer complications. 30

Diagnostic accuracy for CSP/PAS in specialized diagnostic centers, mainly using ultrasound but relying on nuclear magnetic resonance for specific cases, is usually higher than 90%. 31 32 33 Recently, one expert consensus by modified Delphi procedure was published regarding definitions and sonographic reporting systems for CSP 34 and another for assessing the recommended ultrasound signs for evaluation of PAS. 35 According to the latter, ultrasound signs more helpful in predicting surgical outcomes in patients at high risk for PAS are (1) loss of clear zone, (2) bladder wall interruption, (3) placental lacunae and (4) placenta previa involving the cervix. These recommendations should ideally be used to standardize diagnostic features across all centers.

Most importantly, previous PAS classifications are not helpful for diagnostic or treatment purposes since there are many false positives and false negatives when trying to distinguish between accreta/increta/percreta or FIGO 1/2/3 cases 12 36 as well as between uterine dehiscence with placental intrusion and PAS cases, both by imaging studies and intraoperative assessment. 37 Based on current pathophysiological concepts, it seems much more appropriate that imaging specialists should focus on features that would help counsel patients regarding the risks of worse perinatal outcomes, such as those described on the modified Delphi consensus, and help the surgeons regarding the necessary interventions. Fetal medicine specialists should do their best to describe the topography of the uterine dehiscence(s), its size, proximity to other pelvic structures (such as the bladder, cervix, parametria, uterine arteries), degree and location of sub placental vascularity, and lower and upper limits of the placenta. A face-to-face debriefing between the diagnostic and the surgical team before the surgery or a preoperative/intraoperative scan would be highly recommended to help the multidisciplinary team prepare for more challenging cases and define essential strategies, such as type of skin incision, location of hysterotomy, use of ureteral stenting or invasive radiology and the possibility of a sub-total hysterectomy.

**Fig. 2:**
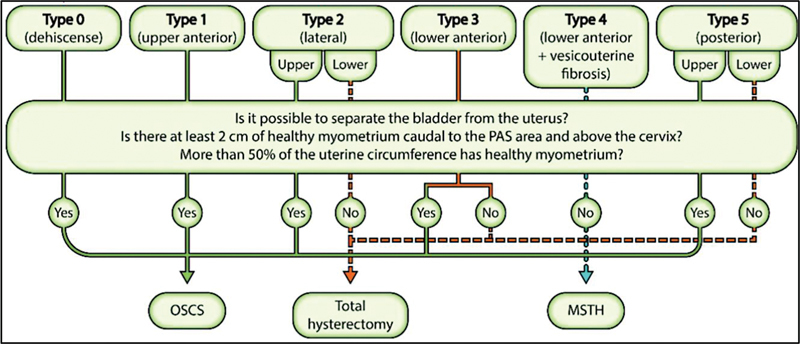
Intraoperative topographical classification for placenta accreta spectrum and management protocol. MSTH, Modified subtotal hysterectomy; OSCS, One-step conservative surgery; PAS, Placenta accreta spectrum. **Source:** Adapted from Nieto-Calvache AJ, Aguilera LR. Simulation, a fundamental component of training to treat placenta accreta spectrum. Rev Bras Ginecol Obstet. 2022;44(12):1159-60. doi: 10.1055/s-0042-1760216. 38

Instead of trying to describe the “depth of invasion/protrusion/involvement”, fetal medicine specialists should do their best to anticipate, as much as possible, uterine, and placental features targeted on the PAS topography classification and surgical staging, which will eventually impact on the management decision, 17 18 as shown in Figure 2 . Intraoperatively, surgeons will analyze three essential aspects: (1) Is it possible to separate the bladder from the uterus? (2) Is there at least 2 cm of healthy myometrium caudal to the PAS area and above the cervix? (3) Is there healthy myometrium in over 50% of the uterine circumference? Positive answers to these 3 questions would direct the surgical team to a more conservative approach, achievable in almost 80% of cases. 18 38 From the point of view of imaging studies, there is lack of evidence to support prediction of bladder-uterus adhesive disease (question 1). However, placental implantation above/below the superior bladder reflection, the thickness of the myometrial layer, the vascularity of the myometrium/bladder interface, and the sliding sign between the bladder and the gravid uterus should be further explored in future studies and might be helpful to predict surgically challenging cases. In addition, the transvaginal scan can accurately define the characteristics of the placenta and the distance of its lower border from the internal cervical os, as well as cervical remodeling (question 2). Finally, the size and location of the uterine dehiscence/PAS should be clearly described, helping to answer question 3.

As previously cited, it may be challenging to differentiate a large uterine dehiscence with overlying placenta from an anterior lower uterine PAS. But does it matter? The definitive diagnosis between PAS vs. non-PAS is the role of a targeted histopathological diagnosis. More than that, even extensive uterine dehiscence might lead to increased bleeding and the need for a hysterectomy, outcomes that could eventually be more severe than in minor PAS cases. 37

Another major issue is that the previously published literature does not report the diagnostic accuracy of prenatal imaging in predicting complex cases of PAS. Most studies reported the prediction of maternal outcome, including need for transfusion or hysterectomy. However, these measures are not entirely associated with difficulty at surgery and are largely affected by several factors, such as operator's experience or type of intervention, and do not necessary reflect the difficulty at surgery. Studies exploring the diagnostic performance of ultrasound in predicting complex case are needed to improve prenatal counselling and management of pregnancies complicated by PAS.

Potential PAS diagnoses issued by expert sonographers must be always confirmed by intraoperative staging and before carrying out potentially morbid interventions. 17 The presence of imaging specialist on the surgical theater is recommended and feedback from the surgical team to the prenatal diagnosis team are essential to ascertain diagnostic quality control and improve the whole team performance.

Antenatal diagnosis of PAS is not easy and most obstetricians do not receive comprehensive training during their residence to diagnose or treat this disease. It is essential to join efforts at the regional and international level to provide women with the best possible care. Telehealth emerges as a strategy to accelerate the set-up of regionalization of care. Telemedicine support can be directed to both primary and specialized services, improving timely diagnosis, promoting individualized and accurate treatment, and strengthening local interdisciplinary groups. 39 40

To conclude, qualified ultrasound screening for CSP/PAS should be widely available, focusing on risk factors and placental position on ultrasound. Referral pathways to PAS specialized centers should be set up to ensure prompt assessment and confirmation of a high-risk PAS situation. Trained providers should assess these pregnant women. All efforts should be made to start the topographical classification of PAS antenatally and assist in the surgical planning, which must be carried out in the specialized referral center to manage PAS.
